# Supplementary material for: Relation between Photogrammetry and Spinal Mouse for Lumbopelvic Assessment in Adolescents with Thoracic Kyphosis
Source: Healthcare (Basel). 2024 Mar 28;12(7):738. doi: 10.3390/healthcare12070738 (PMC11012063; doi:10.3390/healthcare12070738)
Supplement: Supplementary file 1 [file healthcare-12-00738-s001.zip › Figure S1.pdf]

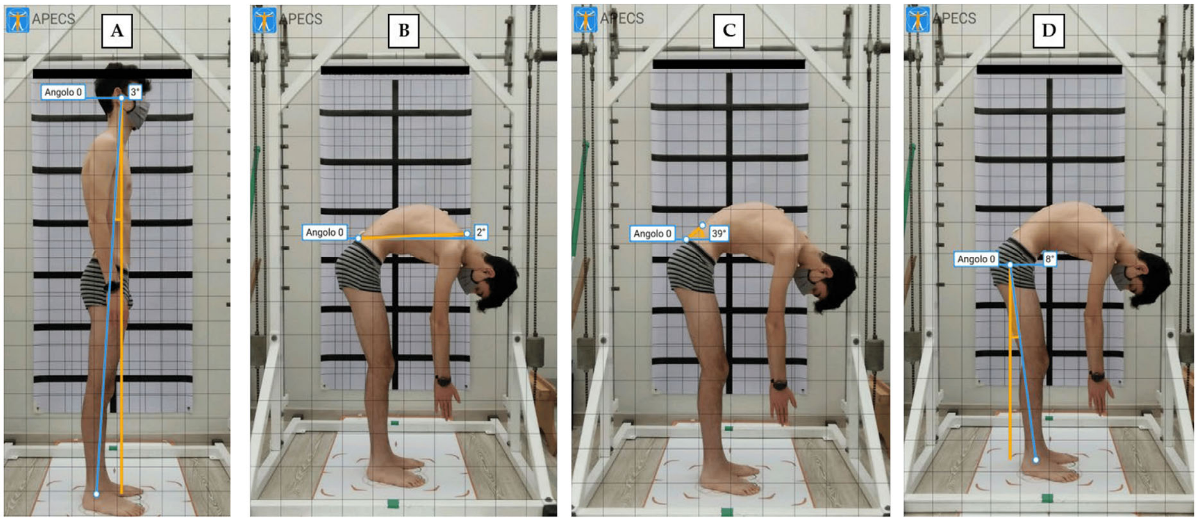

Figure S1. Photogrammetry evaluation in both standing and bending positions. Note: This figure is adapted from Belli et al., 2023 [19].
