# Supplementary figures and images for: Relation between Photogrammetry and Spinal Mouse for Lumbopelvic Assessment in Adolescents with Thoracic Kyphosis
Source: Healthcare (Basel). 2024 Mar 28;12(7):738. doi: 10.3390/healthcare12070738 (PMC11012063; doi:10.3390/healthcare12070738)

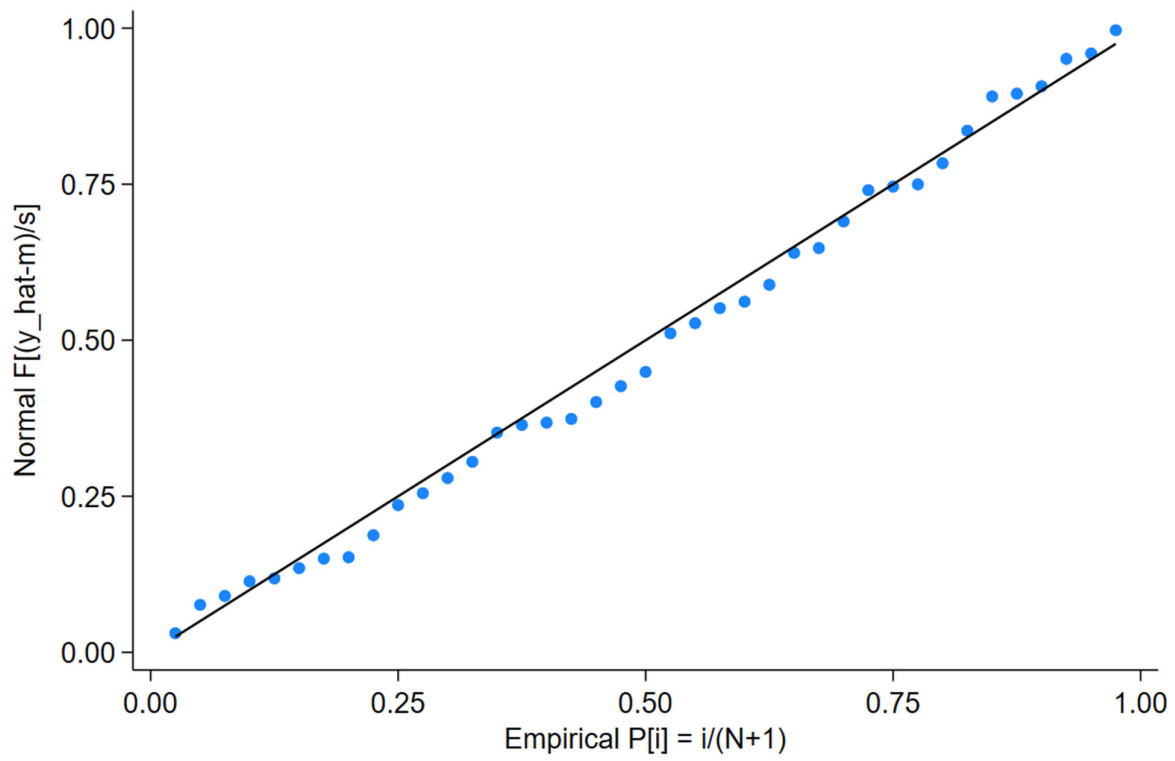

**Figure S2.** Q-Q plot of model residuals.

Supplement: Supplementary file 1 [file healthcare-12-00738-s001.zip › Figure S2.pdf]
